# HMGB1: A Common Biomarker and Potential Target for TBI, Neuroinflammation, Epilepsy, and Cognitive Dysfunction

**DOI:** 10.3389/fnins.2018.00628

**Published:** 2018-09-11

**Authors:** Yam Nath Paudel, Mohd. Farooq Shaikh, Ayanabha Chakraborti, Yatinesh Kumari, Ángel Aledo-Serrano, Katina Aleksovska, Marina Koutsodontis Machado Alvim, Iekhsan Othman

**Affiliations:** ^1^Neuropharmacology Research Laboratory, Jeffrey Cheah School of Medicine and Health Sciences, Monash University Malaysia, Bandar Sunway, Malaysia; ^2^Department of Surgery, University of Alabama at Birmingham, Birmingham, AL, United States; ^3^Department of Neurology, Epilepsy Program, Hospital Ruber Internacional, Madrid, Spain; ^4^Medical Faculty, Department of Neurology, “Saints Cyril and Methodius” University, Skopje, Macedonia; ^5^Department of Neurology, Neuroimaging Laboratory, State University of Campinas, Campinas, Brazil

**Keywords:** HMGB1, RAGE, TLR4, TBI, epilepsy, neuroinflammation, cognitive dysfunction

## Abstract

High mobility group box protein 1 (HMGB1) is a ubiquitous nuclear protein released by glia and neurons upon inflammasome activation and activates receptor for advanced glycation end products (RAGE) and toll-like receptor (TLR) 4 on the target cells. HMGB1/TLR4 axis is a key initiator of neuroinflammation. In recent days, more attention has been paid to HMGB1 due to its contribution in traumatic brain injury (TBI), neuroinflammatory conditions, epileptogenesis, and cognitive impairments and has emerged as a novel target for those conditions. Nevertheless, HMGB1 has not been portrayed as a common prognostic biomarker for these HMGB1 mediated pathologies. The current review discusses the contribution of HMGB1/TLR4/RAGE signaling in several brain injury, neuroinflammation mediated disorders, epileptogenesis and cognitive dysfunctions and in the light of available evidence, argued the possibilities of HMGB1 as a common viable biomarker of the above mentioned neurological dysfunctions. Furthermore, the review also addresses the result of preclinical studies focused on HMGB1 targeted therapy by the HMGB1 antagonist in several ranges of HMGB1 mediated conditions and noted an encouraging result. These findings suggest HMGB1 as a potential candidate to be a common biomarker of TBI, neuroinflammation, epileptogenesis, and cognitive dysfunctions which can be used for early prediction and progression of those neurological diseases. Future study should explore toward the translational implication of HMGB1 which can open the windows of opportunities for the development of innovative therapeutics that could prevent several associated HMGB1 mediated pathologies discussed herein.

## Highlights

- The nuclear protein HMGB1 is a mediator for neurological conditions such as TBI, neuroinflammation, epilepsy and cognitive dysfunction.

- HMGB1 could be a common functional biomarker of TBI, neuroinflammation, epileptogenesis and cognitive dysfunction.

- Inhibiting the HMGB1/RAGE/TLR4 signaling axis could be a novel therapeutic strategy against several HMGB1 mediated conditions like TBI, neuroinflammation, epilepsy and cognitive dysfunction.

## Introduction

Epilepsy is a serious neurological condition characterized by spontaneous and recurrent seizures (Liu et al., 2018) affecting people of all ages. Current anti-epileptic drugs (AEDs) only provides symptomatic relief rather than interfering with the disease mechanism, as well as one third of the patients are resistant to AEDs (Ravizza et al., 2017; Walker et al., 2017). Moreover, epilepsy imposes a burden by impacting several aspects of patients and family life. In addition, the burden is intensified due to the ranges of associated comorbidities such as cognitive dysfunctions, anxiety and depression. Hence, the development of novel biomarker which can predict and assess the disease condition as well as patient’s outcome of the therapy against epilepsy is an unmet clinical need. As well as there is a pressing need of exploring new therapy against epilepsy which not only retard the seizure precipitation but also minimizes the associated comorbidities. In this regard, HMGB1 has emerged as a novel frontier and mounting number of preclinical studies targeting HMGB1 have been successful in diverse ranges of neurological conditions provoked by inflammatory responses (Wang et al., 2017; Zhao et al., 2017; Andersson et al., 2018).

High mobility group box 1 proteins are a family of DAMPs (Lotze and Tracey, 2005), which are highly conserved non-histone nuclear proteins and contributes to the architecture of chromatin DNA (Baxevanis and Landsman, 1995). HMGB1 acts as an inflammatory cytokine in response to epileptogenic insults (Kaneko et al., 2017). HMGB1 acts as a pathogenic inflammatory response to mediate ranges of conditions such as epilepsy (Maroso et al., 2010), septic shock (Wang et al., 1999), ischemia (Kim et al., 2006; Wang et al., 2015), TBI (Okuma et al., 2012), PD (Sasaki et al., 2016), AD (Fujita et al., 2016), and MS (Andersson et al., 2008). Structural evaluation of HMGB1 suggests that it exhibits two domains for DNA-binding, known as box A and box B, as well as C-terminal acidic tail comprised of repeating glutamic and aspartic acid residues (Venereau et al., 2016; Aucott et al., 2018b). DAMPs can influence synaptic function in the brain regions such as the hippocampus, which is involved in hyperexcitability and cognitive decline in epilepsy (Ravizza et al., 2017). It has been reported that immediately after neuronal injury, there is a passive release of significant amounts of HMGB1 from the nucleus into the extracellular space (Scaffidi et al., 2002).

High mobility group box 1 has several extracellular receptors such as RAGE, TLR9, TLR4, TLR2, integrin, a-synuclein filaments, proteoglycans, T-cell immunoglobulin and mucin domain (TIM-3), triggering receptor expressed on myeloid cells-1 (TREM1), cluster of differentiation 24 (CD24), C-X-C CXCR4, *N*-methyl-D-aspartate receptor (NMDAR) (Kang et al., 2014). Among these receptors, RAGE and TLR4 are the only receptors that are extensively studied and reported without doubt (Andersson et al., 2018). HMGB1 initiates several cell responses including inflammation as well as mediate the activation of inflammatory process via binding with RAGE and TLR4 (Bianchi and Manfredi, 2007; Iori et al., 2013). During neuroinflammatory conditions, HMGB1 is actively released by neurons and glia cells upon inflammasome activation and in turn activates at least two PRRs, namely TLR4 and RAGE on target cells (Ravizza et al., 2017). Once released extracellularly, HMGB1 binds to the TLR4 and RAGE expressed by immune cells which leads to nuclear factor kappa-light-chain-enhancer of activated B cells (NF-κB) mediated production of pro-inflammatory cytokines (Iori, 2017). Role of HMGB1 in the development and disease of central nervous system (CNS) has been well described (Fang et al., 2012) where contribution of HMGB1 to the neurogenesis in the early phase of brain development, in neurite extension as well as its dual role in neural development and neurodegeneration is well discussed. HMGB1 promoted neuronal differentiation of adult hippocampal neural progenitors via activation of RAGE/NF-κB suggesting the role of HMGB1 in maintaining and sustaining hippocampal neurogenesis (Meneghini et al., 2013).

A previous study reported HMGB1 translocation as the main culprit for TBI (Li Y. et al., 2017). Recently, HMGB1 has received greater attention for its role in epilepsy (Zhao et al., 2017). It has been hypothesized that HMGB1 might be involved in epileptogenesis, especially through BBB disruption and induction of inflammatory processes though the precise mechanism remains still elusive. Several studies were previously conducted to determine the involvement of HMGB1 in the pathogenesis of epilepsy (Fu et al., 2017). HMGB1 plays a pivotal role in cognitive decline where HMGB1 is supposed to caused disruption of the BBB leading to cognitive deficits in aged rats (He et al., 2012). Interestingly, accumulating evidence suggests that neuroinflammation is highly associated with epilepsy and cognitive dysfunction after TBI and HMGB1 exhibits a key role as an initiator and amplifier of neuroinflammation as well as in neuronal excitation (Frank et al., 2015). Annegers and Coan (2000) suggested that there is a high risk of epilepsy after TBI and epilepsy is associated with neurological comorbidities such as cognitive dysfunction (Pascente et al., 2016; Ravizza et al., 2017).

Treatments based on HMGB1 antagonists via targeting extracellular HMGB1 have generated encouraging results in a wide number of experimental models though the clinical studies are yet to be reported. Anti-HMGB1 monoclonal antibodies (mAbs) have demonstrated beneficial effects on epilepsy and TBI. The therapeutic benefits of anti-HMGB1 mAb on epilepsy have been previously demonstrated in animal model of epilepsy (Fu et al., 2017). Potential neuroprotective effects of HMGB1 has been reported in few studies where anti-HMGB1 mAb prevented intracerebral hemorrhage (ICH)-induced brain injury (Wang et al., 2017).

**FIGURE 1:**
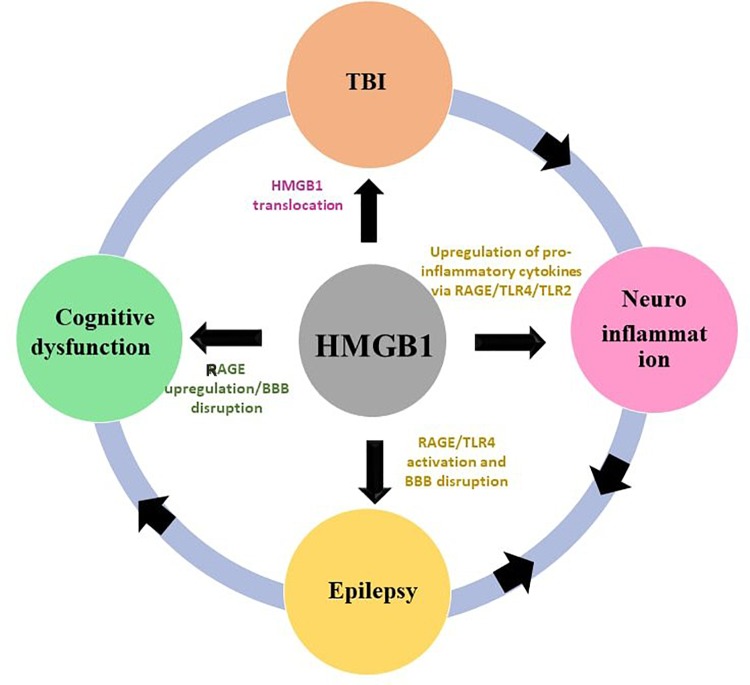
Interlinkage of HMGB1 with TBI, neuroinflammation, epilepsy and cognitive dysfunction. The HMGB1 contributes to the pathogenesis of TBI, neuroinflammation, epilepsy, and cognitive dysfunction through a putative mechanism outlined in this figure.

Interestingly, HMGB1 has been associated with all of the neurological conditions previously outlined (**Figure 1**). Neuroinflammation is the mediator of damage in TBI, epileptogenesis and cognitive decline are the post-TBI events. This makes worth further exploring the HMGB1 and the rationale behind the current review is to explore the potential of HMGB1 as a common biomarker and potential target for several neurological phenotypes discussed in this review. In spite of its proven role in TBI, neuroinflammation, epilepsy and cognitive decline there has been little interest in exploring HMGB1 as a common target and biomarker for those conditions. The preclinical and clinical evidences discussed herein strengthens HMGB1 to stand as a promising candidate to be the common biomarker and treatment target for the neurological conditions where neuroinflammatory pathway plays a central role. Hence, this review summarizes recent advances and discuss these emerging findings to explore the potential of HMGB1 as a common biomarkers and treatment target which could pave the way in developing therapies with broad application modifying the disease progression.

## Neuroinflammation as a Prime Driver of TBI, Epilepsy and Cognitive Decline?

Neuroinflammation is the key component of neuropathology after TBI and contributes to the chronic neurodegeneration and neurological impairments associated with TBI (Kumar and Loane, 2012). This is supported by gene profiling studies which show that genes related to neuroinflammation are upregulated after brain injury (Kobori et al., 2002) and elevated levels of the inflammatory cytokines TNF-α, TGF-β, and IL-1β are expressed after TBI (Morganti-Kossmann et al., 2002; DeKosky et al., 2013). Several experimental and clinical evidences support the role of inflammatory mediators as the origin of both seizures and epileptogenesis (Vezzani and Granata, 2005; Shimada et al., 2014). Brain inflammation contributes to the generation of individual seizures as well as cell death, which in turn contributes to the development of seizures via activation of inflammatory pathways (Vezzani et al., 2011). Moreover, there is evidence which highlights that inflammation can be a cause as well as consequences of epilepsy (Vezzani et al., 2011).

Traumatic brain injury incites a neuroinflammatory axis in the brain which perpetuates neurodegeneration and increases the chances of initiating epileptogenesis (Webster et al., 2017) However, the probability of developing epilepsy after TBI varies from 4.4% to 53% (Frey, 2003) and becomes more likely with a higher injury severity and a younger age at injury (Barlow et al., 2000). Neuroinflammation is a contributing factor to the pathophysiology of post-TBI epileptogenesis (Riazi et al., 2010). Neuroinflammation can alter the BBB permeability directly via cytokine aided activation of metalloproteinase or via disruption of tight junctions (Gloor et al., 2001; Utech et al., 2009), though the precise mechanism remains elusive. It is worthwhile to note that although neuroinflammation is typically provoked after a series of epileptogenic brain injuries, the proportion of patients developing the disease is small (Vezzani, 2015). The pivotal role of neuroinflammation in cognitive dysfunction has been reported but the underlying molecular mechanism are not yet known. However, there are insights regarding certain inflammatory pathways underlying hyperexcitability and excitotoxicity that can promote cognitive decline (Vezzani, 2015).

In recent days, research using experimental and clinical models has focused on the pathogenesis of how HMGB1 proteins contributes to TBI, neuroinflammation, epilepsy and cognitive decline. Such research has sought to pave the way to understand how these mechanisms can be interfered to develop therapies for the aforementioned neurological conditions. Moreover, accumulating evidence reported beneficial effects on evaluating anti-HMGB1 mAb and HMGB1 inhibitors against TBI, neuroinflammation, epilepsy, and cognitive decline. We will therefore discuss the outcomes of such experimental and clinical experiments in an individual pattern.

## Role of HMGB1 in TBI

TBI is an insult to the brain through any external mechanical force (Webster et al., 2017), which makes TBI a devastating and intractable cause of worldwide morbidity and mortality. Survivors live the rest of their lives with cognitive, motor, behavioral or speech and language disabilities (Richard et al., 2017). However, the pathophysiology of TBI is still elusive and a tremendous research must be made to explore the progression of neurodegeneration and the ensuing inflammatory processes (Parker et al., 2017). It is currently unavailable to attenuate the pathological process of TBI and improve neurological deficits (Jiang et al., 2018). TBI involves a primary insult known as structural damage due to any external mechanical force which is followed by a secondary injury including a multitude of neuroinflammatory phenomena such as excitotoxicity, oxidative stress and apoptosis (Webster et al., 2017). These processes begin within minute after TBI and can persist for months to years and is suspected to contribute to the expansion of tissue damage (Hinson et al., 2015).

**FIGURE 2:**
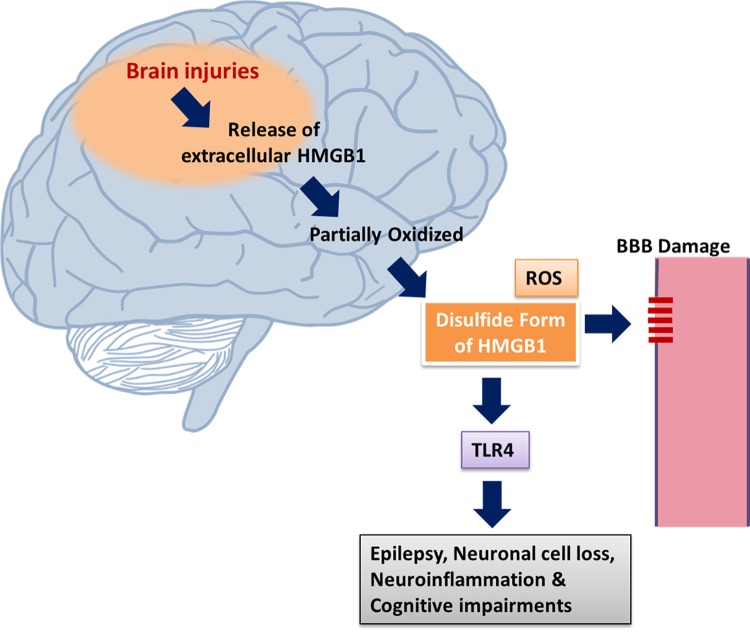
Mechanism of HMGB1 release via brain injury. HMGB1 translocation results from brain injuries and epileptogenesis. Dying cells, neurons and glia are responsible for the release of extracellular HMGB1 which can be partially oxidized. Neuronal excitability is enhanced via the mediation of pro-inflammatory activities through the activation of TLR4 signaling by disulphide HMGB1. The stabilization of HMGB1 in its disulphide form is promoted by the generation of ROS. HMGB1 via activating receptors RAGE and TLR4 leads to neuronal cell loss, neuroinflammation, epilepsy, and cognitive dysfunction (Ravizza et al., 2017).

During TBI, HMGB1 is released via the *N*-methyl D-aspartate receptor subtype 2B (NR2B)-mediated mechanism from necrotic neurons (Richard et al., 2017). HMGB1 mediates sterile inflammation and provokes macrophages and endothelial cells to release TNF-α, IL-1, and IL-6 by binding with RAGE and TLR4. This binding further activates the NF-κB pathway and facilitates the upregulation of HMGB1 and the expression of pro-inflammatory mediators (Gao et al., 2012). In addition, neuroinflammatory processes mainly mediated by activated microglia and astrocytes are crucial for the initiation and progression of TBI (Li D. et al., 2017). TBI induces a series of events including BBB breakdown, brain edema, upregulation of tight junction proteins (TJPs), expression of inflammation related molecules (Yang et al., 2018). TLR4 has been linked with TBI where TLR4 mediates glial phagocytic activity and inflammatory cytokines production (Jiang et al., 2018) and plays an important role in inflammatory response and brain injury (Fang et al., 2013). Once HMGB1 is released into the extracellular settings following TBI, it binds to transmembrane major mediators of the inflammatory response, TLR2, TLR4, and RAGE (Yang et al., 2005). Excessive inflammation resulting from activation of the HMGB1/TLR4 pathway in the brain has been implicated in TBI and ischemia-reperfusion injury (Yang et al., 2011). However, the mechanistic interlinkage between intracellular danger signaling, which involves the nuclear chromatin-binding factor, HMGB1 and inflammatory pathways after TBI is not yet fully understood (Parker et al., 2017). There is an increased understanding that TBI may induce activation of HMGB1/TLR4/RAGE/NF-κB signaling pathway and inflammatory cytokine expression, which would induce and/or aggravate the secondary brain injury where HMGB1 is supposed to implicate a critical role in promoting inflammation and aggravating damage after TBI (Xiangjin et al., 2014) (**Figure 2**). Several HMGB1 inhibitors have demonstrated protective effect against TBI via inhibiting HMGB1/TLR4/NF-κB pathway activation (Su et al., 2011), and by reducing HMGB1/RAGE interaction (Okuma et al., 2014).

Inhibition of HMGB1 expression and the TLR4/NF-κB pathways exhibits protective effects in animal model of TBI. HMGB1 inhibitors (glycyrrhizic acid) attenuated TBI by inhibiting the classically activated microglia/macrophages (M1) phenotype activation and promoting the alternatively activated microglia/macrophages (M2) phenotype activation of microglia/macrophages, via the inhibition of HMGB1 and suggest that targeting of HMGB1 to modulate the microglia/macrophage polarization might be a potential approach for TBI (Gao et al., 2018). Similar line of results has been reported where anti-HMGB1 mAb improved neurological deficits in ICH-induced brain injury. Anti-HMGB1 mAb inhibited the release of HMGB1 into the extracellular space in the peri-hematomal region, reduced serum HMGB1 levels and decreased brain edema by protecting BBB integrity, in association with decreased activated microglia and the expression of inflammation-related factors at 24 h after ICH (Wang et al., 2017). Neuroprotective effects of TLR4 knockdown has emerged as a promising approach for TBI. TLR4 knockdown ameliorated neuroinflammatory response and brain injury after TBI and suppressing autophagy induction and astrocyte activation is postulated the main mechanism behind the neuroprotective effects of TLR4 (Jiang et al., 2018). Data obtained from western blot analysis in an experimental study reported the release profile of HMGB1 and RAGE after TBI where HMGB1 was released as soon as 30 min after TBI and a decline in its expression was noted between 1 and 6 h after TBI. However, the expression level of RGAE was elevated at 6 h after TBI and reached its peak after 1 day (**Table 1**) (Gao et al., 2012). An immunostaining study reported that septic brain injury results in increased HMGB1 cytoplasmic translocation in neurons (**Table 1**) (Li Y. et al., 2017). A few studies have reported a noteworthy elevation of HMGB1, IL-1β, and TNF-α levels in serum as estimated by an enzyme linked immunosorbent assay (ELISA) kit in an experimentally induced TBI model in rabbits (Mohamed et al., 2017). However, ELISA analysis does not precisely differentiate between release pattern of HMGB1 either by necrosis, or from macrophages and monocytes, or a combination of both (Au et al., 2012).

**Table 1:** Summary of findings reporting HMGB1 in TBI.

S.N.	Intervention	Model	Mechanism	Observations	Reference
1.	HMGB1	Rat	• Inhibition of HMGB1 expression and TLR4/NF-κB pathway	↓ Reduced expression of HMGB1 and TLR4	Su et al., 2011
				• Improved motor function and lessened brain oedema	
2	Anti-HMGB1 mAb	Rat	• Protection against BBB disruption	• Inhibition of translocation of HMGB1, protection of BBB permeability	Okuma et al., 2012
			• Inhibition of the inflammatory responses		
				• Downregulation of inflammatory molecule expression	
				• Improvement of motor function	
3	HMGB1	Rat	• Interference with HMGB1 and RAGE interaction	• Inhibited the ↑ in BBB permeability and impairment in motor functions	Okuma et al., 2014
			• Inhibition of the expressions of TNF-a, IL-1β, and IL-6		
				• Inhibition of translocation of HMGB1 in neurons at the site of injury	
4	Anti-HMGB1 mAb	Rat	• Protecting BBB integrity	• ↓ Release of HMGB1 to the extracellular space in the peri-hematomal region	Wang et al., 2017
			• ↓ Expression of inflammation-related factors		
				• ↓ Serum HMGB1 levels and brain edema through maintaining BBB integrity	
5	HMGB1	Rat	• Downregulation of sepsis-induced RAGE and NF-κBp65 expression	• HMGB1was ↑ in the cytoplasm via translocation	Li Y. et al., 2017
				• RAGE and NF-κβ p 65 were up regulated after brain injury	
				• HMGB1 and its signaling transduction have a key role in the pathogenesis of septic brain injury	
6	HMGB1	Human	• Targeting HMGB1/RAGE signaling	• HMGB1 disappeared or translocated from the nucleus to the cytoplasm at early stages after TBI	Gao et al., 2012
				• RAGE expression ↑ after TBI	
7	HMGB1	Human	• Activation of microglial TLR4 and the subsequent expression of AQP4	• Peak CSF HMGB1 level in human TBI was within 0–72 h.	Laird et al., 2014
				• HMGB1 released from necrotic neurons through a NR2B-mediated mechanism	

*S.N., serial number; ↑, increased; ↓, decreased; HMGB1, high mobility group box 1; TRL4, Toll like receptor 4; RAGE, receptor for advanced glycation end products; NF-κB p 65, nuclear factor kappa-light-chain-enhancer of activated β cells p 65; IL-6, interleukin-6; IL-1β, interleukin-1β; TNF-α, tumor necrosis factor-α; BBB, blood–brain barrier; TBI, traumatic brain injury; CSF, cerebrospinal fluid; MRI, magnetic resonance imaging; ICP, intra-cranial pressure; NR2B, *N*-methyl D-aspartate receptor subtype 2B; AQP4, astrocytic water channel aquaporin-4*.

High mobility group box 1 A-box fragment, an antagonist competing with HMGB1 for receptor binding, significantly ameliorated the BBB breakdown and brain edema induced by controlled cortical impact (CCI), and these effects were associated with the decrease in expressions of inflammation-related factors as well as improved neurological functions (Yang et al., 2018). Ethyl pyruvate (**Table 1**) (Su et al., 2011) and omega-3 polyunsaturated fatty acid supplementation (Chen et al., 2017b) has demonstrated its effectiveness against TBI via inhibition of the HMGB1/TLR4/NF-κB pathway. Evaluation of anti-HMGB1 mAb therapy against TBI in rats reported that anti-HMGB1 mAb remarkably inhibited fluid percussion-induced brain edema in rats, which was associated with an inhibition of HMGB1 translocation, protection of BBB architecture, downregulation of inflammatory molecule expression, and improvement in motor function (**Table 1**) (Okuma et al., 2012). Increased inhibition of the expression of HMGB1 signaling axis, with RAGE and TLR4, NF-κB DNA binding and downstream inflammatory cytokines were reported on glycyrrhizin treatment (Okuma et al., 2014).

The literature review ranging from human to animal studies suggests an important association between TBI and increased levels of HMGB1 in serum and cerebrospinal fluid (CSF) (Okuma et al., 2014). In addition, emerging data reported HMGB1 in the CSF of subarachnoid hemorrhage (SAH) (Nakahara et al., 2009) and in the serum of ICH (Zhou et al., 2010) highlighting as a potential biomarker of neurological outcome. Nevertheless, the clinical output of HMGB1 antagonist against several forms of brain injury is yet to be reported. Similar line of evidence were observed in which HMGB1 is associated with increased levels of intracranial pressure (ICP) in patients and promoted cerebral edema after TBI where the detrimental effects of HMGB1 are mediated through the microglial TLR4 activation and the expression of the astrocytic water channel aquaporin-4 (AQP4) (Richard et al., 2017). HMGB1 plasma levels were reported to increase within 30 min after severe trauma in humans and suggested a correlation between plasma levels of HMGB1 with early post-traumatic coagulopathy and severe systemic inflammatory response (Cohen et al., 2009). There was a 30-fold increment of plasma HMGB1 levels after trauma, as compared to normal controls during a 1-h period of injury which provides insights regarding the post-injury elevation levels of HMGB1 in human (Peltz et al., 2009) However, the study did not report any correlation between HMGB1 levels and the patients’ outcome. Furthermore, a compelling relationship between plasma HMGB1 absorption and the severity of acute TBI was unraveled and correlation between Glasgow Coma Scale score and HMGB1 levels were reported, which can serve as prognostic information in patients with severe TBI (Wang et al., 2012). Higher CSF HMGB1s level are considered as an important biomarker to predict outcome after pediatric TBI (Au et al., 2012).

The pathophysiology behind the complex inflammation cascades secondary to TBI is not yet fully understood. However, resulting injuries and outcomes after TBI have been studied in the past and have suggested HMGB1 to be a major player in disease progression as well as a potential therapeutic target to reduce the injuries and improve outcome following TBI. As well as the current biomarkers of TBI such as glial fibrillary acidic protein (GFAP) and S100B (Vos et al., 2010) are limited by low sensitivity, predictivity, and specificity (Metting et al., 2012). In this regard, due to its profound role in TBI pathology and inflammatory pathways post-TBI, HMGB1 appears to be a promising candidate which can be used as a prognostic marker of TBI. Moreover, downregulating HMGB1/RAGE/TLR4/NF-κB axis might be a novel strategy against TBI which could attenuate the neurological functions as well.

## Role of HMGB1 in Neuroinflammation and Related Pathologies

Neuroinflammation is considered as an innate immune responses in the CNS, which is triggered in response to several inflammatory signals such as pathogen infection, injury or trauma, which might ultimately result in neurotoxicity (Streit et al., 2004). Microglia are known as the predominant innate immune cell in the CNS and are thus considered a pivotal mediator of neuroinflammatory processes (Gehrmann et al., 1995). Microglia express TLRs, in particular TLR4 (Ransohoff and Perry, 2009), and are reported to mediate the pro-inflammatory effects of HMGB1 in peripheral innate immune cells (Yang et al., 2010). Disulphide form of HMGB1 (ds-HMGB1) potentiates the microglia pro-inflammatory response to an immune challenge suggesting that acute increases or exposure to ds-HMGB1, as may occur during acute stress or trauma, might induce a primed immune phenotype in the CNS, which may lead to an exacerbated neuroinflammatory response if exposure to a subsequent pro-inflammatory stimulus occurs (Frank et al., 2016). There is an increased understanding that HMGB1 mediates inflammatory and immune reactions in CNS and emerging evidence reveals that HMGB1 plays an essential role in neuroinflammation through receptors such as TLR, RAGE, and NMDAR (Wan et al., 2016). Moreover, HMGB1 induces RAGE and TLR4 mediated neuroinflammation and necrosis after injuries such as lesions in the spinal cord and brain (Fang et al., 2012). HMGB1 mediates inflammation by activating the innate immune receptors during sterile injury, in a similar manner to activation by PAMPs (Yang and Tracey, 2010). The BBB is a special microvessel structure in the CNS, consisting of microvascular endothelial cells sealed by tight junctions. Its permeability is closely related with degeneration, injury and inflammation of the CNS (Huang et al., 2011). Similar line of study shows that damaged BBB correlates directly with neuroinflammation involving microglial activation and reactive astrogliosis, which is associated with increased expression and/or release of HMGB1 (Festoff et al., 2016). In an experiment evaluating the contribution of extracellular, cerebral HMGB1 (in absence of other DAMPs) in its disulphide or fully redox form to neuroinflammation demonstrate that ds-HMGB1 and fully redox HMGB1 (fr-HMGB1) function as pro-inflammatory mediators in the CNS, promoting BBB disruption and cytokine production (Aucott et al., 2018a). Thus, anti-neuroinflammation and maintenance of BBB integrity may be potential targets for neuroprotection (Cheng et al., 2018). Increasing evidence suggests that selective targeting of CNS inflammation is a viable strategy for interfering disease onset or progression for a number of neurodegenerative disorders where neuroinflammation is the key player (Hong et al., 2016). Despite the deteriorating role of neuroinflammation in many neurological diseases, the number of existing anti-inflammatory drugs is quite limited because of insufficient efficacy or undesired side effect (Craft et al., 2005). HMGB1 has emerged has a novel frontiers due to its plausible role in neuroinflammation (Lee et al., 2014) as well as in inflammatory diseases (Harris et al., 2012) where the causal role for HMGB1 in a range of non-degenerative neuroinflammatory conditions has been well reported. Moreover, HMGB1 blocking therapies have proven to be highly beneficial, demonstrating remarkable neuroprotection in several neuroinflammation models (Kim et al., 2006).

Inflachromene (ICM), a microglial inhibitor possessing anti-inflammatory effects via binding with HMGB1 blocks the sequential processes of cytoplasmic localization and extracellular release of HMGBs by perturbing its post-translational modification as well as downregulates pro-inflammatory functions of HMGB and reduces neuronal damage *in vivo* demonstrating its potential against neuroinflammatory diseases (Lee et al., 2014). HMGB1 binds with lipopolysaccharides (LPS) and IL-1 to initiate and synergize TLR4-mediated pro-inflammatory response and immediately after pro-inflammatory stimulation by LPS, TNF-α, IL-1, IL-6, and IL-8, HMGB1 is released from activated monocytes and macrophages (Youn et al., 2008). The regulation of HMGB1 secretion is crucial for the regulation of HMGB1 mediated inflammation and is dependent on various processes such as phosphorylation by calcium-dependent protein kinase C (Oh et al., 2009). HMGB1 acts as a novel pro-inflammatory cytokine-like factor and regulates excitotoxicity-induced acute damage processes and delayed inflammatory mechanisms in the post-ischemic brain of Sprague Dawley (SD) rats (**Table 2**) (Kim et al., 2006). Elevation of HMGB1 in brain was measured in several non-degenerative neuroinflammatory condition such as ethanol exposure (Zou and Crews, 2014), and stress-induced neuroinflammatory priming (Weber et al., 2015). Neuroinflammation contributes to the progression of several neurodegenerative diseases including PD (Tansey and Goldberg, 2010) and AD (Heneka et al., 2015). Blocking the neuroinflammatory pathways in these neurodegenerative diseases will exerts neuroprotection against these diseases. Anti-HMGB1 mAb has inhibited the activation of microglia, prevents BBB breakdown, and inhibit the expression of inflammation cytokines such as IL-1β and IL-6 in an experimental model of PD demonstrating its neuroprotective effects possibly via suppressing neuroinflammation (Sasaki et al., 2016). Glycyrrhizin attenuated neuroinflammation, cognitive deficits, microglial activation related over-expression of pro-inflammatory cytokines in the hippocampus induced by LPS showcasing its therapeutic potential against neurodegenerative diseases like AD (Song et al., 2013).

**Table 2:** Summary of findings reporting HMGB1 in neuroinflammation mediated conditions.

S.N.	Intervention	Model	Mechanism	Observation	Reference
1.	HMGB1	Rat	• Delayed inflammatory processes by extracellular HMGB1	• HMGB1 was released during the excitotoxicity-induced acute damaging process	Kim et al., 2006
				• Extracellular HMGB1 provokes inflammatory processes and acts like a novel pro-inflammatory cytokine-like factor	
2	HMGB1	Mice	• Activation of NF-κB and NADPH oxidase by HMGB1 via binding with Mac1	• HMGB1-Mac1-NADPH oxidase signaling cascades connects chronic neuroinflammation and dopaminergic neurodegeneration	Gao et al., 2011
3	HMGB1	Rat	• HMGB1 acted as an early pro-inflammatory cytokine	• HMGB1 released into the cytoplasm soon after ICH	Lei et al., 2013
				• Mediate inflammation during the acute phase of ICH	
4	HMGB1	Rat	• Inflammatory responses produced via HMGB1/TLR4/NF-κB signaling	• HMGB1 ↓ the release of IL-6 and TNF-α	Tian et al., 2015
				• HMGB1 inhibited activation of NF-κB in the developing brain	
5	HMGB1	Rat	• Regulation of age-related priming of the neuroinflammatory responses by HMGB1	• HMGB1was ↑ in aged rodent brains and CSF	Fonken et al., 2016
				• Blocking HMGB1 “desensitized” microglia in the aged brain and prevent pathological infection-elicited neuroinflammatory responses	

*S.N., serial number; ↑, increased; ↓, decreased; HMGB1, high mobility group box 1; ICH, intracerebral hemorrhage; Mac1, macrophage antigen complex 1; CSF, cerebrospinal fluid; TNF-α, tumor necrosis factor-α; NF-κB, nuclear factor kappa-light-chain-enhancer of activated B cells; TLR4, Toll like receptor 4; RAGE, receptor for advanced glycation end products; IL-6, interleukin-6; NADPH, nicotinamide adenine dinucleotide phosphate hydrogen*.

Multiple sclerosis is an autoimmune-mediated chronic, inflammatory, demyelinating disease of CNS characterized by axonal damage (Compston and Coles, 2008). Experimental autoimmune encephalomyelitis (EAE) is the most reliable experimental model of MS (Miller et al., 2010). HMGB1 is receiving increasing attention in autoimmune disorders including MS. The very first study unraveling the role of HMGB1 in the pathophysiology of MS reported increased numbers of macrophages with cytoplasmic HMGB1 in active lesions (Andersson et al., 2008) and suggest HMGB1 as a novel biomarker of inflammatory demyelinating disease. Several researches has emerged on the base of earlier studies and suggest that the expression and release of HMGB1 are remarkably elevated in several stages of EAE where HMGB1 expression pattern is dynamically changed during the progression of EAE, as well as validated HMGB1 as a key mediator of EAE pathology (Sun et al., 2015). Targeting HMGB1 locally might exhibits therapeutic potential against EAE which can attenuate the disease severity and incidence as well as delayed disease onset time (Robinson et al., 2013). Neutralization of HMGB1 appears to be a novel strategy against MS as evidenced by an experimental study of MS, where anti-HMGB1 mAb ameliorated clinical severity, reduced CNS pathology, and blocked the production of pro-inflammatory cytokine (Uzawa et al., 2013).

Amyotrophic lateral sclerosis (ALS) is a non-demyelinating neurodegenerative disease characterized by increased neuronal loss and enhanced neuroinflammation, with extensive activation of glial cells and microglia stimulation releasing pro-inflammatory molecules, ROS, and nitric oxide (Ray et al., 2016). Neuroinflammation is postulated as a pathological hallmark of ALS (Lewis et al., 2012), and HMGB1 has been extensively studied in ALS due to its putative involvement in the pathology of ALS which is elusive yet. However, the elevated level of HMGB1 in the spinal cord of transgenic mice (SOD1G93A transgenic mice) were observed and reported that HMGB1 may have a role in the progressive inflammatory and neurodegenerative processes in response to the neurotoxic environment present in the spinal cord of SOD1G93A mice rather than to be involved as a primary event in the motor neuron death (Coco et al., 2007).

Withaferin A in an animal model of HMGB1-induced inflammatory responses suppressed the production of IL-6, TNF-α and the activation NF-κB by HMGB1 (Lee et al., 2012). HMGB1 acts a pathogenic factor in many inflammatory conditions including experimental arthritis models (Schierbeck et al., 2011). HMGB1 has been observed to be a key mediator of intestinal inflammation in non-alcoholic fatty liver disease (NAFLD) via RAGE and redox signaling (Chandrashekaran et al., 2017). Another study reported that liver inflammation in diabetic mice was improved via regulation of the HMGB1/TLR4/NF-κB signaling pathway (Yin et al., 2018). Taken together, these results clearly highlight HMGB1 as a key mediator in several inflammatory diseases and suggest that HMGB1 exhibits therapeutic potential against these HMGB1 mediated inflammatory disease. Evaluating the ameliorative effects of glycyrrhizin on SAH in a rat model significantly improved neurological scores, reduced HMGB1-positive cells, downregulated mRNA and protein levels of HMGB1, inhibited BBB permeability, and attenuated neuronal cell death and apoptosis after SAH, suggesting it as a promising candidate for brain inflammation (Ieong et al., 2018).

The result obtained from human studies on MS patients corroborated with the experimental studies where HMGB1 and its receptors (RAGE, TLR2, and TLR4) were up-regulated in CSF of MS patients implicating that RAGE, TLR2, and TLR4 actively participate in an inflammatory, innate immune response driving and shaping the ensuing adaptive immune response during MS (Andersson et al., 2008). In clinical studies in patients with ALS, activation of TLR/RAGE signaling pathways were observed as evidenced by the elevated expression of HMGB1 and its receptors in reactive glia in human ALS spinal cord. The activation of these pathways might contribute to the progression of inflammation, resulting in motor neuron injury (Casula et al., 2011). In addition, serum HMGB1 auto antibody (Ab) has been suggested as a biomarker for the diagnosis of ALS and can be used to monitor disease progression (Hwang et al., 2013). High level of HMGB1, IL-6, and IL-17A has been detected in CSF of patients with an anti-NMDA receptor (NMDAR) encephalitis (neuroinflammatory disorder) (Ai et al., 2018), reflecting the underlying neuroinflammatory processes but does not report any precise role of HMGB1 in disease pathology. Clinical study performed on patients with AD and mild cognitive impairment (MCI) observed enhanced BBB permeability by HMGB1 and suggest HMGB1 as a clinical biomarker as well as validates HMGB1 as a non-invasive biomarker of BBB dysfunction and neuroinflammation which can assess the progression of neurodegeneration in AD and MCI patients (Festoff et al., 2016).

On the ground of evidences highlighted above, HMGB1 has been implicated in ranges of neuroinflammatory diseases as well as inflammation mediated disorders and HMGB1 exhibits huge potential to be a reliable biomarker for neuroinflammation related pathologies. On the positive note, beneficial effects of targeting HMGB1 in brain inflammation related pathologies is well documented. Extensive exploration to innovate therapeutic strategy to attenuate uncontrolled neuroinflammation triggered by HMGB1 is a pressing need.

## Role of HMGB1 in Epilepsy

**FIGURE 3:**
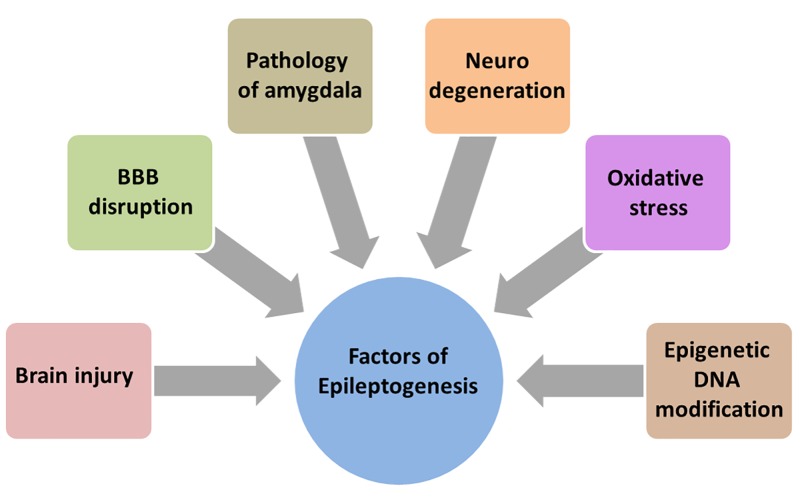
Array of factors contributing to epileptogenesis. Much is unknown about the precise mechanism of epileptogenesis making it difficult to design and develop new therapies. But there is an increased understanding that brain injury, BBB disruption, neurodegeneration, oxidative stress, epigenetic DNA modification and pathology of amygdala contribute and aggravate the epileptogenesis.

Epileptogenesis is described as complex structural changes in the brain that convert a normal brain into a brain debilitated by recurrent seizure activity (Sloviter and Bumanglag, 2013). Neurodegeneration (Pitkanen and Lukasiuk, 2009; Reddy, 2013), disruption of BBB (Bar-Klein et al., 2017), the amygdala (Aroniadou-Anderjaska et al., 2008), the glutamatergic system (Aroniadou-Anderjaska et al., 2008), oxidative stress (Ashrafi et al., 2007), and epigenetic modification of DNA (Hauser et al., 1993) are all involved in epileptogenesis and aggravates the process (**Figure 3**).

Due to the lack of disease modifying effect in mainstream AEDs, precise understanding of diseases pathology and developing novel therapeutic approach for epilepsy-related hyperexcitability is a current need. Searching for molecular mediators of epileptogenesis in animal models, much attention has been paid to the potential pathogenic role of HMGB1 in the generation and recurrence of seizures (Kaneko et al., 2017; Yang et al., 2017). In spite of tremendous advancement in research, the pathogenesis of epilepsy is still complex, however, brain inflammation is supposed to play the role (Riazi et al., 2010). There is mounting evidence which report that neuroinflammatory processes in the pathophysiology of seizures/epilepsy and HMGB1 were found to behave like an inflammatory cytokine in response to epileptogenic insults (Kaneko et al., 2017). Glial cells activation has been reported to serve an important role in the development of epilepsy and HMGB1 may mediate microglial activation via the TLR4/NF-κB signaling pathway during seizures (Shi et al., 2018). HMGB1 activates IL-1R/TLR signaling in neurons and has a key role in seizure generation and recurrence via rapid sarcoma family kinases catalyzed phosphorylation of NMDA-NR2B receptors (Vezzani et al., 2012). As well as, HMGB1 serves a key role in epileptogenesis via microglial activation, via TLR4-NF-κB signaling pathway activation (Shi et al., 2018). An array of investigation has reported the role of HMGB1 in seizure but the precise mechanism on how HMGB1 leads to seizure generation is not documented well. HMGB1 released from glia and neurons and its signaling with TLR4 are suggested in generating and perpetuating seizures, the suggestion was based on the anti-convulsant activity of TLR4 inhibitors and Box A, a competitor of endogenous HMGB1 but the study lacks detailed mechanism how HMGB1/TLR4 axis leads to seizure generation (Maroso et al., 2010). Although HMGB1 activates both TLR4 and RAGE, the role of RAGE in seizures is less prominent than that of TLR4 (Iori et al., 2013).

Mesial temporal lobe epilepsy (MTLE) is a most common refractory focal epilepsy syndromes (Palleria et al., 2015) and role of HMGB1 in the pathogenesis of MTLE remains unknown. Experimental MTLE study reported significant upregulation of HMGB1 and TLR4 gene expression in the hippocampi of a rat and correlated this overexpression of HMGB1 and TLR4 to the pathogenesis of MTLE in immature rats (Yang et al., 2017). In addition, the role of HMGB1 and its receptors (RAGE and TLR4), including the pro-inflammatory cytokine IL-1β, in generating and perpetuating seizures is well documented (Zaben et al., 2017). Pharmacological and genetic studies on animal and clinical brain specimens showed that translocation and release of HMGB1 occurs in the pathological epileptogenic focus of different type of epilepsy (Maroso et al., 2010; Iori et al., 2013). It is worth noting that HMGB1/TLR4 axis not only reduced seizure frequency and duration, but also accelerated seizure onset, which usually occurs within minutes in kainite and bicuculline-induced seizure models implicating the important role of HMGB1 in the precipitation of the first seizure after a pro-convulsant administration (Maroso et al., 2010). Abnormal extracellular HMGB1 might contribute to the pathophysiology of epilepsy-related hyperexcitability as evidenced by study on PRNCs demonstrate a surge in extracellular HMGB1 approximated seizure initiation, establishing HMGB1 as a key pathophysiological contributor to the onset of epilepsy-related hyperexcitability (**Table 3**) (Kaneko et al., 2017).

**Table 3:** Summary of findings reporting HMGB1 in epilepsy.

S.N.	Intervention	Model	Mechanism	Observations	Reference
1	HMGB1	KA-induced seizure in mice	• Targeting HMGB1/TLR4 axis	• ↑ Frequency of seizure and total duration • Seizure can be ↓ by TLR4 and HMGB1 antagonists	Maroso et al., 2010
2	Anti-HMGB1 mAb	Acute seizure (MES and PTZ); Chronic seizure by KA in mice	• Inhibition of HMGB1 translocation	• ↓ Seizure threshold; ↓ time in tonic–clonic seizures and ↓ death • Delayed onset of generalized seizures; ↓ seizure stage; ↓ incidence of tonic seizures	Zhao et al., 2017
3	Molecular isoforms of HMGB1	Electrically induced Se in rats	• Activation of HMGB1/TLR4 axis	• ↑ level of HMGB1 and its acetylated and disulphide isoforms in blood	Walker et al., 2017
4	HMGB1	Pilocarpine-induced SE in rats	• Regulation of P-gp expression via RAGE/NF-κB inflammatory signaling pathways	• ↓ The expression levels of MDR1A/B mRNA and P-gp protein	Xie et al., 2017
5	HMGB1	KA-induced epilepsy in rats	• Modulation of glutamate metabolism	• ↑ Extracellular HMGB1 suggesting contribution of HMGB1 in epilepsy related hyperexcitability • Translocation of HMGB1from nucleus to cytosol after KA administration	Kaneko et al., 2017
6	HMGB1	Pilocarpine-induced epilepsy in rats	• Targeting HMGB1 via TLR4/NF-kB signaling pathway	• Inhibit the development of AE-related epilepsy • Suppression of HMGB1 expression • MiR-129-5p mediated TLR4/NF-kB signaling pathway ameliorated AE-related epilepsy	Liu et al., 2017
7	Anti-HMGB1 mAb	Pilocarpine-induced SE in mice	• Inhibition of HMGB1 release and inflammation	• Protection of BBB permeability; ↓ HMGB1 translocation • ↓ Latency and frequency of stage 5 seizures	Fu et al., 2017
8	Molecular isoforms of HMGB1	Human	• Evaluation of HMGB1 isoforms as mechanistic biomarkers of epileptogenesis in sera obtained from epileptic patients	• HMGB1 isoforms in the brain and blood were changed • Expression of disulphide HMGB1 in newly diagnosed epilepsy patients	Walker et al., 2017

*S.N., serial number; ↑, increased; ↓, decreased; BBB, blood–brain barrier; Pgp, *P*-glycoprotein; MES, maximal electroshock seizures; KA, kainic acid; PTZ, pentylenetetrazol; HMGB1, high mobility group box 1; EEG, electroencephalogram; MDR1A/B, multidrug resistance protein 1A/B; RAGE, receptor for advanced glycation end products; NF-κB, nuclear factor kappa-light-chain-enhancer of activated B cells; miR-129-5p, micro RNA-129-5p; qRT-PCR, quantitative real-time polymerase chain reaction; AE, autoimmune encephalitis; mAb, monoclonal antibody; PRNCs, primary rats neural cells*.

TLR4 activation in neurons and astrocytes by HMGB1 proteins is a key mechanism of seizure generation and blocking TLR4 signaling using an antagonist could also reduce the severity of epilepsy (Iori et al., 2013). Investigation on post-surgery patients with intractable epilepsy revealed increased levels of HMGB1, TLR4, RAGE, NF-κB, p65 and inducible nitric oxide synthase (iNOS) in the brain of the epilepsy group as well as increased levels of IL-1, IL-6, TNF-α, TGF-β, and IL-10 in epilepsy patients (Shi et al., 2018).

**FIGURE 4:**
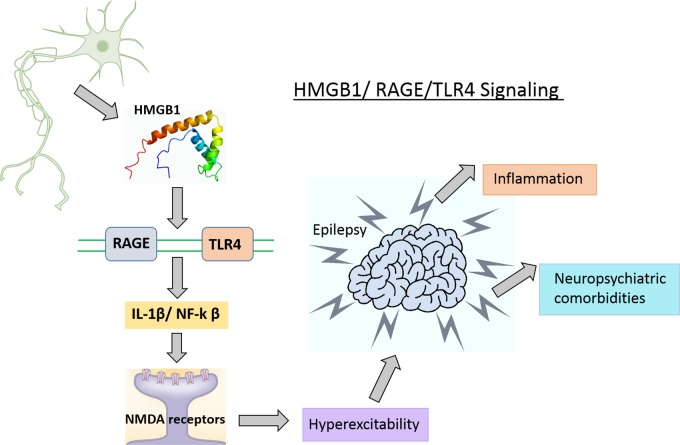
HMGB1/TLR4/RAGE signaling in epilepsy. HMGB1 released from glia and neuronal cells in the CNS, activates its main receptor (TLR4 and RAGE) and contributes to epileptogenesis possibly via BBB disruption and activation of neuroinflammatory pathways.

Targeting HMGB1/TLR4/RAGE signaling for epilepsy has gained more attention in recent years. MicroRNA-129-5p inhibited the development of autoimmune encephalomyelitis (AE)-related epilepsy by HMGB1 expression and inhibiting the TLR4/NF-κB signaling pathway (Liu et al., 2017). HMGB1 and TLR4 antagonists slowed seizure precipitation, prevented acute and chronic seizure recurrence in C57BL/6 mice as well as reported increased expression of HMGB1 and TLR4 in human epileptogenic tissue, which is similar to a mouse model of chronic seizures and suggest a role for the HMGB1-TLR4 axis in human epilepsy (Maroso et al., 2010). Thus, HMGB1/RAGE/TLR4 signaling might contribute toward the generation and perpetuation of seizures (**Figure 4**) in humans and can be successfully targeted to attain anti-convulsant effects in epilepsies which are resistant to drugs. The expression level of HMGB1 was significantly elevated in the hippocampus and cortex after 24-h in a KA-induced model of SE, which suggests that the HMGB1 protein has a key role in epilepsy (Walker et al., 2014). HMGB1 enhances hypothermia induced seizures and contributes to the pathogenesis of febrile seizure. However, the precise mechanism of HMGB1 in febrile seizures remains unclear (Ito et al., 2017).

Limited data is available regarding evaluation of therapeutic benefits of HMGB1 inhibitors in animal models of epilepsy. However, glycyrrhizin demonstrates neuroprotection against lithium/pilocarpine-induced SE in rats, as well as ameliorates pilocarpine-induced oxidative injury and inflammatory responses via suppressing IL-1β and TNF-α, (González-Reyes et al., 2016) but did not demonstrate anti-epileptic activity. Dynamic changes in HMGB1 expression in the hippocampus of the mouse brain was reported after KA administration and glycyrrhizin exerts neuroprotective but not anti-epileptic effects via suppressing both acute and delayed HMGB1 inductions in the hippocampal cornu ammonis (CA)1 and CA3 region as well as its accumulation in serum (Luo et al., 2014).

Anti-HMGB1 mAb demonstrated an anti-seizure effect as evident by the lack of a disruption on the physical EEG rhythm and basic physical functions as it prevents the translocation of HMGB1 from nuclei following a seizure. This anti-seizure effect was not observed in TLR4 knockout mice (**Table 3**) (Zhao et al., 2017). Moreover, anti-HMGB1 mAb also demonstrated a disease-modifying anti-epileptogenic effect on epileptogenesis after SE, as evidenced by a reduced seizure frequency and improved cognitive function (Zhao et al., 2017). The minimization of seizure frequency and duration can be achieved by inhibitors of HMGB1 which is supposed to act via targeting the HMGB1/RAGE/TLR4 axis and retard seizure precipitation via inhibition of HMGB1 translocation, protection of BBB integrity. In a similar line, anti-HMGB1 mAb exhibited inhibitory effects on the BBB leakage and pilocarpine-induced HMGB1 translocation. As well as prevented the BBB permeability and reduced HMGB1 translocation (**Table 3**) (Fu et al., 2017). Zhao et al. (2017) evaluated the anti-epileptic effect of anti-HMGB1 mAb on human brain slices from clinical drug-resistant epilepsy patients (**Table 3**) where anti-HMGB1 mAb binds to HMGB1 and demonstrate long-lasting anti-epileptic properties, which is consistent with the previously estimated long half-time elimination in the brain (Zhang et al., 2011).

Extensive research highlights the putative role of HMGB1 in seizure generation, increased expression level of HMGB1 in epileptic brain (Chen et al., 2015) retardation of seizure precipitation by HMGB1 inhibitors (Zhao et al., 2017) implicating that HMGB1 is involved in all aspect from seizure generation to seizure retardation making HMGB1 as a strong candidate to be a reliable biomarkers for epileptogenesis. Moreover, the prevailing prognostic markers for seizure recurrence and seizure remission in patients diagnosed with epilepsy are solely based on supplementary factors including age, seizure type, EEG, and MRI, but are limited in their accuracy (Ravizza et al., 2017). Earlier study recommended HMGB1 isoforms as a mechanistic biomarkers for epileptogenesis where they investigate the value of blood HMGB1 in predicting epilepsy development as well as differentiating epileptogenic from non-epileptogenic rats after SE (Walker et al., 2017). HMGB1 as a biomarker of epileptogenesis will eventually provide a deeper insight on the normal biologic processes, pathogenic processes, or responses to an exposure or intervention, including therapeutic interventions with broad applications that are clinically able to arrest disease progression or to improve its clinical course. Moreover, precise understanding of mechanistic pathway on how HMGB1 induce seizure via inflammatory signaling will play a vital role in designing new therapies targeting inflammatory pathways to minimize seizures. However, the association between HMGB1 and seizure needs further exploration. Overall findings suggest that blocking the HMGB1/TLR4/RAGE regulatory axis may represent a novel method for treating epilepsy.

## Role of HMGB1 in Cognitive Dysfunction

Cognition refers to a collection of cognitive phenomenon such as learning and memory, attention, executive function, consciousness, and language (McAfoose and Baune, 2009). Cognitive dysfunction is among the most prevalent and debilitating features highly associated with epilepsy (de Krom, 2006), PD (Kalia, 2018), AD (Elgh et al., 2006). The precise mechanism of cognitive dysfunction is not well understood, though there is an increased understanding about chronic activation of cytokine-dependent inflammatory signaling contributing to neuronal dysfunctions manifesting as cognitive deficits (Cunningham and Sanderson, 2008). In addition, increased brain cytokine signaling impairs learning and memory (Dantzer et al., 2008). Moreover, neuroinflammation has been reported to cause memory impairments as evidenced by an experimental study where LPS administration cause memory impairment via inducing neuroinflammation (Lee et al., 2008). Several study has been reported where cognitive impairment has been ameliorated via alleviating neuroinflammation (Ganai and Husain, 2018).

The main focus of the topic is HMGB1, which is an initiator and amplifier of neuroinflammatory process. HMGB1 has been implicated in impairing memory via mediating RAGE and TLR4 (Mazarati et al., 2011) however, the exact mechanism of HMGB1 in cognitive decline is limited. HMGB1 exhibits pro-excitatory effects in the hippocampus by elevating the phosphorylation of NR2B-NMDA receptors (Maroso et al., 2010), and thus increasing the receptor calcium channel conductance (Viviani et al., 2003). NR2B-containing NMDA receptors prevent cell surface expression of the GluR1 subunit of the α-amino-3-hydroxy-5-methyl-4-isoxazole propionic acid (AMPA) receptor (Kim et al., 2005), which has a key role in both synaptic plasticity and memory (Sanderson et al., 2008), including the NORT (Schiapparelli et al., 2006). In the adult brain, NR2B decreases the span of retrovirus-associated DNA sequences (Ras)/extracellular signal-regulated kinases (ERK) activation pathway (Kim et al., 2005), which might also contribute to memory impairment (Weeber and Sweatt, 2002). Remarkably, the same mechanism that modulates the seizure-facilitating effect of HMGB1 (i.e., the activation of NR2B-containing NMDA receptor) might be simultaneously involved in facilitating learning deficits. Recombinant HMGB1 impaired memory encoding in wild type (WT), TLR4 knockout and RAGE knockout animals, but no effect was observed either on memory consolidation, nor retrieval. Moreover, memory deficits was not exhibited on TLR4 knockout nor RAGE knockout mice *per se*. Blockade of TLR4 in RAGE knockout mice using *Rhodobacter sphaeroides* LPS attenuated the memory function impaired by HMGB1(Mazarati et al., 2011). The upregulation of microglia and systemic HMGB1 levels were correlated with cognitive dysfunction (Terrando et al., 2010). IL-1 modulation has been implicated to ameliorate LPS-induced cognitive dysfunction, however, IL-1 blockade ameliorated cognitive decline by reducing microglia without affecting HMGB1 (Terrando et al., 2010).

Plausible detrimental effects of HMGB1 on memory may have broad clinical implications. In an experimental model of chronic cerebral hypoperfusion (CCH), HMGB1 neutralization attenuates hippocampal neuronal death and cognitive impairment where anti-HMGB1 neutralizing Ab exerts long-time positive effects on hippocampal CA1 neuronal survival and cognitive abilities in the chronic phase of CCH as well as preserves BBB integrity, and suppresses hippocampal glial activation, pro-inflammatory cytokine production (Hei et al., 2018). Anti-HMGB1 mAb has ameliorated the symptoms and phenotype of AD in an experimental model where mAb against HMGB1 completely rescued cognitive impairment in a mouse model via inhibiting neurite degeneration even in the presence of amyloid beta (Aβ) plaques. The recovery in the memory impairment was evidenced by Y-maze test (Fujita et al., 2016).

Post-operative cognitive dysfunction is probably the most frequent type of postoperative cognitive impairment and the pathophysiology of POCD remains incompletely understood (Grape et al., 2012). HMGB1 has been extensively studied against POCD. Possible role of neuroinflammation mediated by HMGB1, RAGE, and S100B (a class of DAMPs) was hypothesized in the pathophysiology of POCD, however, the relationship between HMGB1 or S100B or RAGE signaling and cognitive dysfunction was not completely confirmed (Li et al., 2013). HMGB1 and RAGE levels were remarkably upregulated after surgery and HMGB1 is supposed to cause cognitive decline via breaking BBB permeability, however, study did not conclude either BBB is disrupted after surgery and relationship between HMGB1 and cognitive decline cannot be ascertained as the study did not selectively block HMGB1 using mAb (He et al., 2012). Administration of endogenous HMGB1 proteins produced cognitive decline in mice and neutralized HMGB1 mAb ameliorated cognitive decline and inhibited the inflammatory response after tibial surgery, suggesting a initiating role for this mediator in POCD (**Table 4**) (Vacas et al., 2014). As well as clinical data obtained from patients undergoing gastrointestinal surgery showed that serum HMGB1 and IL-6 levels was elevated post-surgery, and the increased post-operative HMGB1 and IL-6 levels were associated with the cognitive decline the occurs 1-week post-surgery (**Table 4**) (Lin et al., 2014). Oral pretreatment of glycyrrhizin inhibited HMGB1 cytosolic expression, alleviates the surgery-Induced HMGB1 upregulation in the hippocampus of the mice and attenuated the severity of post-operative memory impairment, as evidenced by the shorter swimming latency and distance in MWM trials (Chen et al., 2017a). The therapeutic benefits of HMGB1 have been explored in sepsis survivors where HMGB1 mediates cognitive dysfunction in a murine model of severe sepsis survivors (Chavan et al., 2012). Administration of neutralizing anti-HMGB mAb to survivors, beginning 1 week after the onset of peritonitis, significantly ameliorate memory impairments and brain pathology.

**Table 4:** Summary of findings reporting HMGB1 in cognitive dysfunction.

S.N.	Intervention	Model	Mechanism	Observation	Reference
1	HMGB1	Mice	• Activation of inflammatory pathways by stimulating RAGE and TLR4	• ↑ Brain levels of HMGB1 induce cognitive abnormalities and are mediated by either TLR4 or RAGE.	Mazarati et al., 2011
2	HMGB1	Mice	• Activation and trafficking of circulating bone marrow-derived macrophages to the brain	• POCD can be prevented by minimizing the effects of HMGB1 • A neutralizing antibody to HMGB1 protein reduced memory dysfunction	Vacas et al., 2014
3	HMGB1	Mice	• Neuroinflammation mediated by HMGB1 and RAGE	• Expression of HMGB1, RAGE and NF-κB p6 ↑ after surgery. • HMGB1 and RAGE signaling modulate the hippocampal inflammatory response	Koeth et al., 2013
4	HMGB1	Rat	• HMGB1 and RAGE signaling	• HMGB1 and RAGE ↑ in the hippocampus of operated animals • HMGB1 interrupt and regulate the inflammatory response associated with the pathogenesis of POCD	He et al., 2012
5	HMGB1	Human	• Interaction between APOE-ε4 and HMGB1	• HMGB1 showed an association with ↓ cortical thickness • APOE-ε4 and HMGB1 are responsible for extensive cortical thinning in MCI.	Foo et al., 2017
6	HMGB1	Human	• POCD induced via inflammatory response	• HMGB1, IL-6 levels ↑ in patients after surgery • Elevated levels of HMGB1, IL-6 might be associated with cognitive dysfunctions after surgery	Lin et al., 2014

*S.N., serial number; ↑, increased; ↓, decreased; HMGB1, high mobility group box 1; TRL4, toll like receptor 4; RAGE, receptor for advanced glycation end products; NF-κB, nuclear factor kappa-light-chain-enhancer of activated B cells; TEM, transmission electron microscopy; IHC, immunohistochemistry; MCI, mild cognitive impairments; APOE-ε4, Apo lipoprotein E ε4; IL-6, interleukin-6; POCD, post-operative cognitive dysfunctions*.

Cognitive decline induced by epilepsy is supported by number of previous studies (Kundap et al., 2017). Earlier studies documented selective deficits in memory encoding in TLE (Schwarze et al., 2009). The plausible role of HMGB1 in epilepsy induced cognitive dysfunction has not yet been reported, though administration of anti-HMGB1 mAb in mice delayed epilepsy onset as well as ameliorated cognitive functions (Zhao et al., 2017). However, the precise role of HMGB1 protein in epielpsy induced cognitive dysfunction has not yet been reported. In order to obtain a precise understanding, it is necessary to perform a longitudinal studies to investigate the levels of HMGB1 in epileptic animal models and concurrently undertake behavioral studies to assess the cognitive function of the animals and evaluate the expression pattern of HMGB1 throughout the study.

There are very few clinical studies that have been conducted till date regarding the effectiveness of anti-HMGB1 mAb on ameliorating cognitive dysfunction in patients. Foo et al. (2017) earlier reported that an interaction between HMGB1 and APOE-ε4 is associated with cortical thinning in MCI. This interaction was observed by studying genomic DNA extracted from peripheral blood and the plasma HMGB1 was measured with an ELISA kit (**Table 4**) (Foo et al., 2017). In human brain slice studies obtained from surgical resection of clinical drug-resistant epilepsy patients, anti-HMGB1 mAb demonstrated an attenuation of cognitive function as well as a disease-modifying anti-epileptogenesis effect, which is indicated by reduction in seizure frequency (Zhao et al., 2017).

## HMGB1: Translational Implication

Recent investigation shed more light on multiple roles of HMGB1 in a diverse range of pathologies such as brain injury, epilepsy, and neuroinflammation and cognitive decline. Treatments based on HMGB1 antagonists via targeting extracellular HMGB1 have generated encouraging results in a wide number of experimental models of aforementioned HMGB1 mediated pathologies, though the clinical studies are yet to be reported. However, complex biology of HMGB1 has not been fully understood yet and there is a notion that association between HMGB1 and brain injury, epilepsy, neuroinflammation mediated pathologies and cognitive decline requires deeper exploration, as the precise mechanism of on how HMGB1 mediates these neurological conditions are yet to be well documented. Despite of that, the identification of HMGB1 inhibitors results in significant experimental and clinical interest. Moreover, HMGB1 as a common biomarker of TBI, neuroinflammation, epileptogenesis and cognitive decline might be instrumental in assessing the disease progression, early prediction of disease as well as evaluating patient’s response to therapy. Translational implication of HMGB1 will be a paradigm shift, which will not only overcome the limitation of currently available AEDs, improve the cognitive decline as well. Moreover, via inhibiting the neuroinflammatory pathways HMGB1 can ameliorate several brain injuries and neuroinflammation mediated pathologies. More precisely, inhibiting HMGB1/RAGE/TLR4 pathway represents a promising approach which can interfere with disease progression in epileptogenesis, neuroinflammatory disease, several forms of brain injury as well as memory impairment. The focus of the topic is TBI, neuroinflammation, epilepsy and cognitive decline, however, blocking HMGB1 might achieves significant neuroprotection in several forms of neurodegenerative disorders where neuroinflammation plays a crucial role. Future strategy should be focused on exploring several HMGB1 antagonist which can efficiently interact with the main HMGB1-receptor, RAGE, acting as competitive antagonists of HMGB1, such as recombinant box A (the truncated N-terminal domain of HMGB1) or S100P-derived RAGE peptide (Musumeci et al., 2014).

## Summary Of Findings and Conclusion

Neuroinflammation has been implicated in ranges of neurological disorders such as TBI, epilepsy and memory impairment. HMGB1 being the mediator of neuroinflammation has been reported to play crucial role in TBI, neuroinflammatory diseases and epileptogenesis via an unknown mechanism. As well as elevated level of HMGB1 in serum and CSF has been observed in these neuroinflammation mediated pathologies. These strengthens the rationale of our study in suggesting HMGB1 as a common biomarker in TBI, neuroinflammation, epilepsy and cognitive decline. Biomarker discovery together with investigations into novel therapeutic candidates would give a noteworthy headway in the treatment of TBI, epilepsy, memory impairment and neuroinflammation via acting on its mechanistic pathway rather than symptomatic control. In current review, an attempt was made to connect the dots between HMGB1 and its putative role in several forms of brain injury, neuroinflammation mediated conditions, epilepsy and cognitive decline using preclinical and clinical evidence.

Several important limitations regarding the topic should not be ruled out, such as feasibility and viability issues in making HMGB1 a common functional biomarker for neuroinflammation mediated pathologies discussed herein. Can inhibiting HMGB1/RAGE/TLR4 axis be a common target for these neurological conditions? Can therapeutic outcomes obtained from experimental evidence regarding the role of HMGB1 in all these neurological conditions be easily translated into clinical settings? These are the concerns that remains unsolved as more experimental data are yet to come.

Despite accumulating scientific evidence of HMGB1 in the neuroinflammation mediated conditions discussed in current review, no attempt has been made in portraying HMGB1 as common biomarker and target for these HMGB1 mediated neurological conditions. We suggest, HMGB1 proteins can be considered as a promising non-invasive, common biomarker of TBI, neuroinflammation, epilepsy and cognitive dysfunction as it meets many criteria to stand as a common biomarker. It is relatively stable in blood and can be rapidly and inexpensively measured in blood. Moreover, changes in the total HMGB1 levels in the brain during neurological conditions discussed herein, can be mirrored in the blood.

As a concluding remark, drawing evidence from earlier preclinical and clinical studies, the current review advances the concept of positioning HMGB1 as common functional biomarker that can significantly improve risk assessment, diagnosis and monitoring of the neurological diseases discussed in this review. As well as HMGB1 can emerge as a novel avenues against TBI, neuroinflammation, epilepsy and cognitive deficits which acts by blocking the neuroinflammatory pathway.

## Author Contributions

YP and MS carried out literature review, conceptualized, designed and drafted the manuscript. AC, YK, ÁA-S, KA, MA and IO provided critical revisions and contributed to the final manuscript. YK also designed the figures. All authors read and approved the final manuscript.

## Conflict of Interest Statement

The authors declare that the research was conducted in the absence of any commercial or financial relationships that could be construed as a potential conflict of interest.

## Abbreviations

AD: Alzheimer’s disease
APOE-ε4: Apo lipoprotein E-ε4
BBB: blood–brain barrier
BDNF: brain derived neurotropic factor
BLA: basolateral amygdala
COX-2: cyclooxygenase-2
CREB: CAMP response element binding
CXCR4: chemokine receptor type 4
DAMPs: damage-associated molecular patterns
DNA: deoxyribonucleic acid
EEG: electroencephalogram
GABA: gamma amino butyric acid
GEPRS: genetically epilepsy prone rats
GLUR2: glutamate receptor 2
GRIA2: glutamate receptor ionotropic AMPA 2
HMGB1: high mobility group box 1
IHC: immunohistochemistry
KA: kainic acid
KIR4.1: inwardly rectifying potassium channel 4.1
LTP: long-term potentiation
MiR-129-5p: micro RNA-129-5p
MS: multiple sclerosis
MWM: Morris water maze
NORT: novel object recognition test
PAMPs: pathogen-associated molecular patterns
PD: Parkinson’s disease
POCD: post-operative cognitive dysfunction
PRNCs: primary rats neural cells
PRR: pattern recognition receptor
PTZ: pentylenetetrazol
RAGE: receptor for advanced glycation end Products
RAS: retrovirus-associated DNA sequences
ROS: reactive oxygen species
SE: status epilepticus
TBI: traumatic brain injury
TEM: transmission electron microscopy
TGF-β: transforming growth factor-β
TLE: temporal lobe epilepsy
TLR4: toll-like receptor 4
TNF-α: tumor necrosis factor-α.
